# Supplementary figures and images for: Structure of the host cell recognition and penetration machinery of a Staphylococcus aureus bacteriophage
Source: PLoS Pathog. 2020 Feb 18;16(2):e1008314. doi: 10.1371/journal.ppat.1008314 (PMC7048315; doi:10.1371/journal.ppat.1008314)

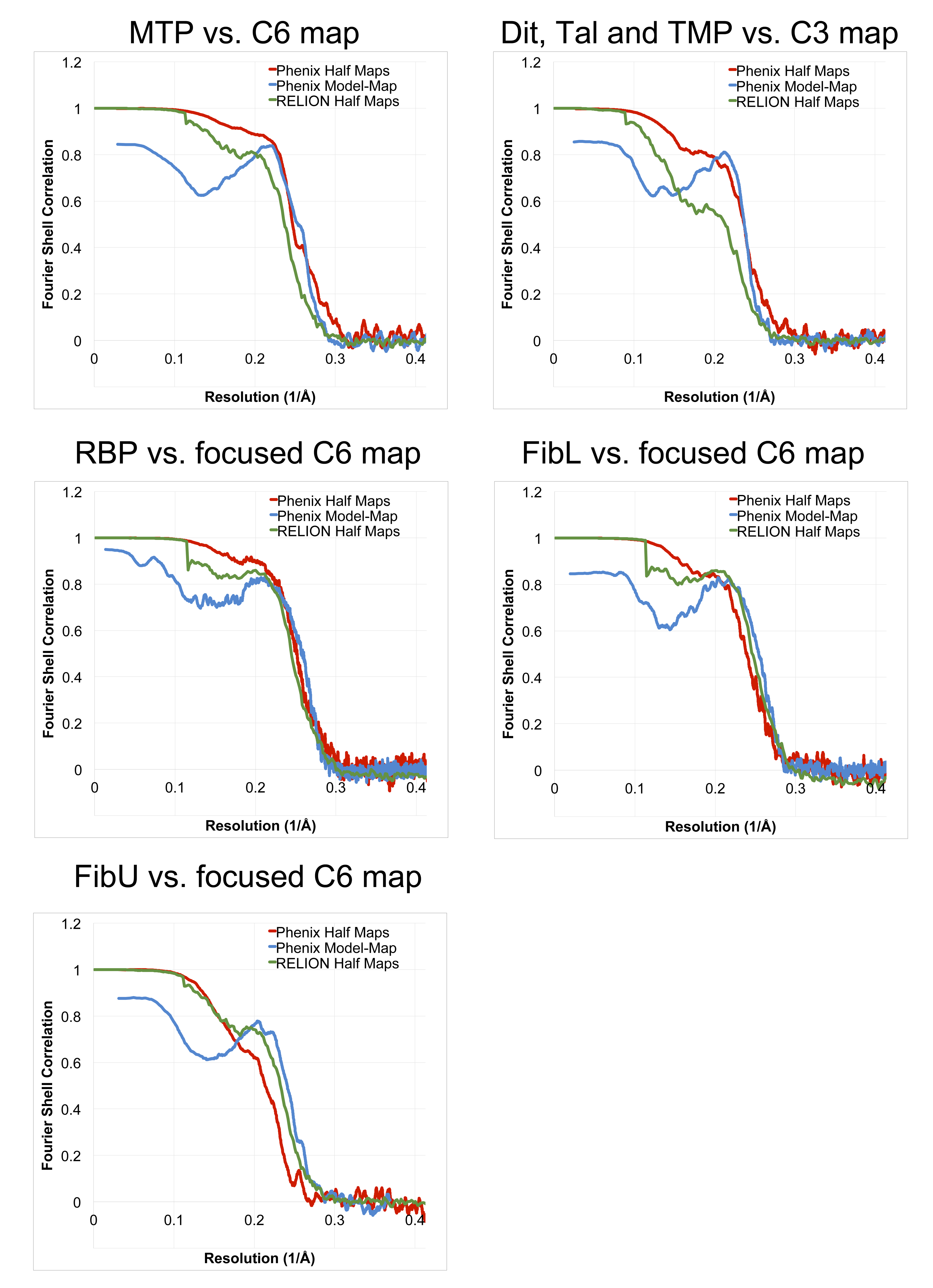

Supplement: S1 Fig — For each refinement, the gold-standard half-map FSC plots for the appropriate maps, calculated in RELION (green curve) and in PHENIX (red curve) are shown. The blue curve represents the corresponding model-to-map FSC plots, calculated in PHENIX. (TIF) [file ppat.1008314.s001.tif]

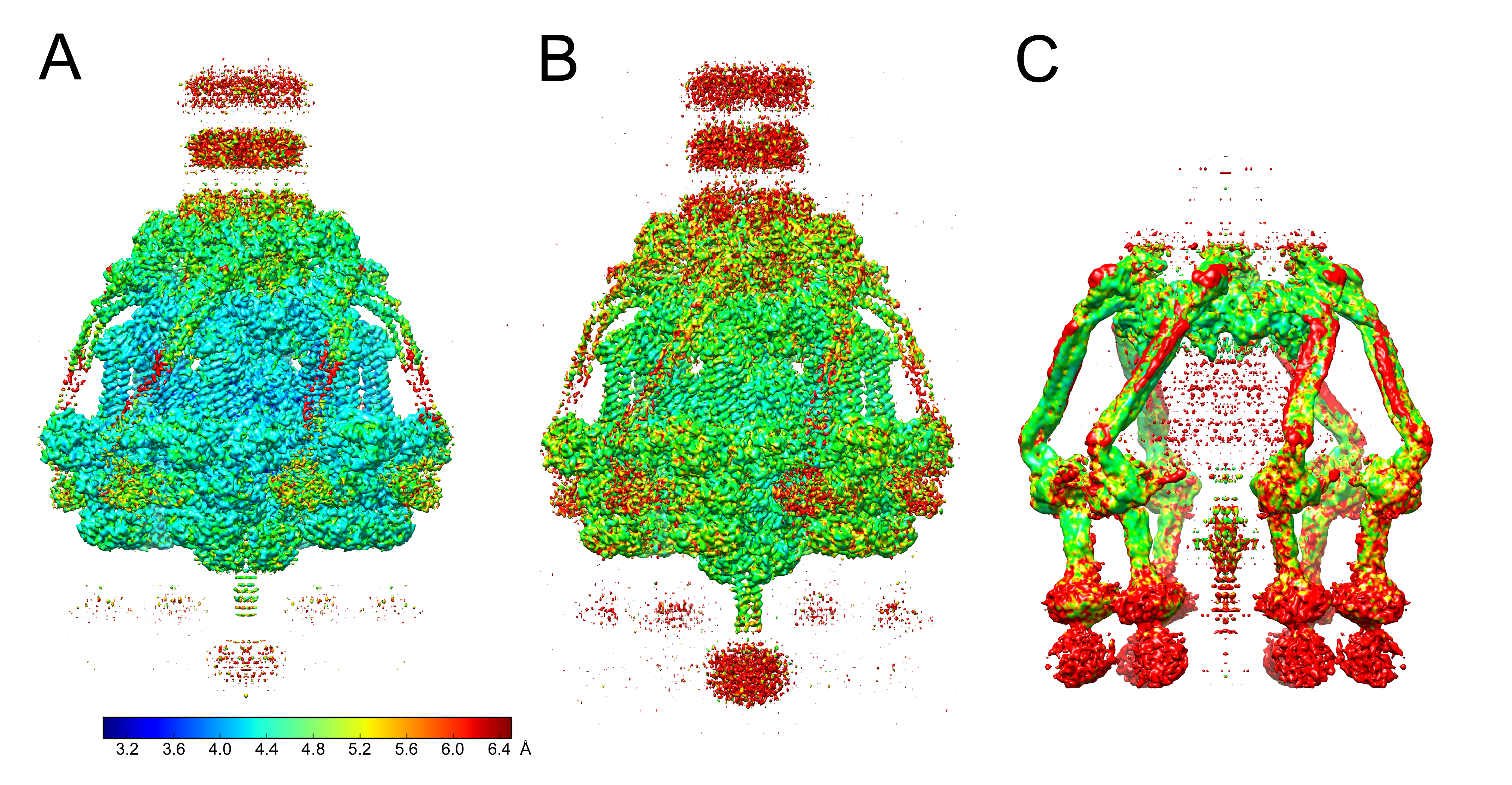

Supplement: S2 Fig — ResMap analysis for the C6 symmetrized map (A), the C3 map (B) and the C6 signal-subtracted map used to model FibL (C). Map resolution is shown from 3.0 Å (blue) to 6.5 Å (red) according to the color bar. (TIF) [file ppat.1008314.s002.tif]

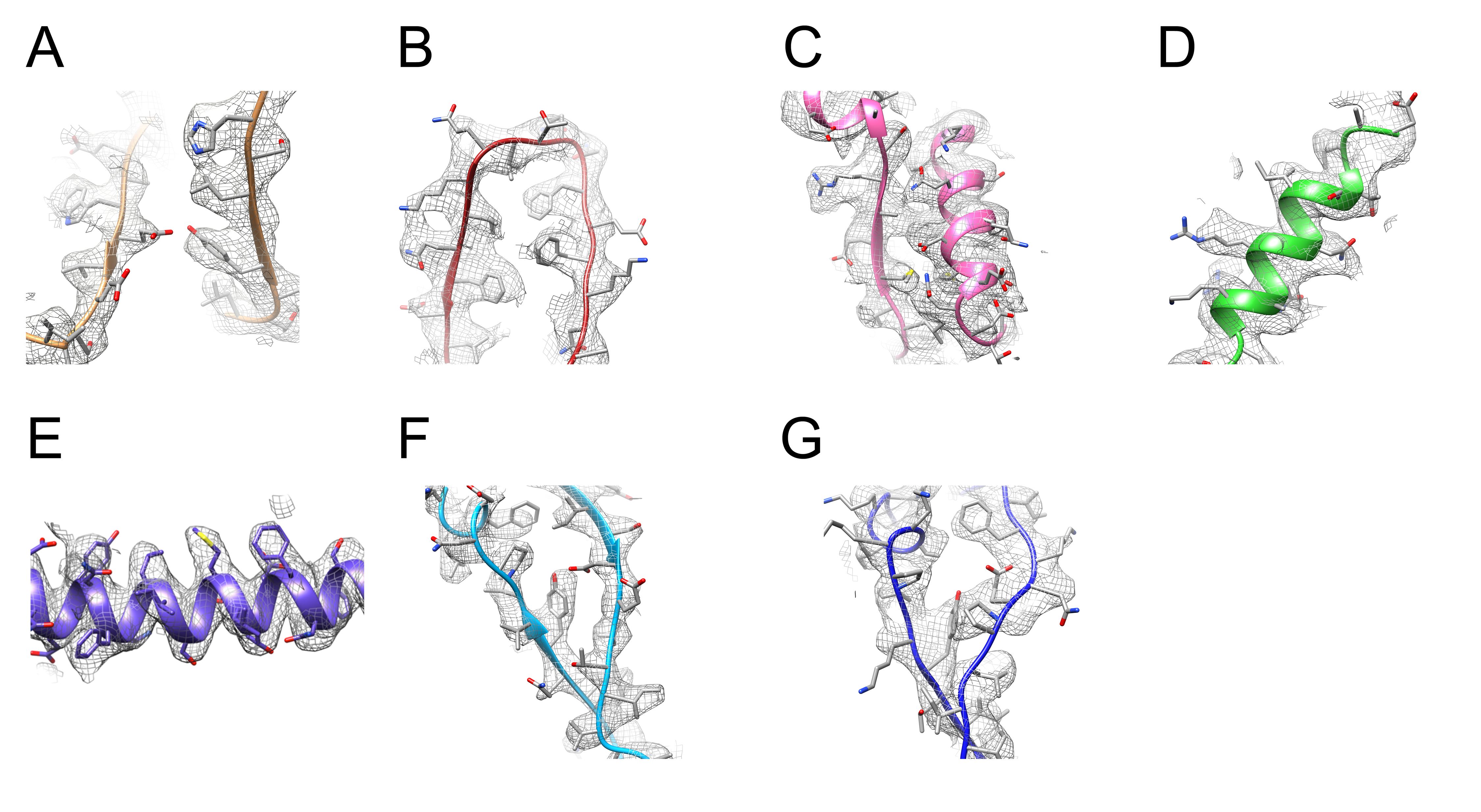

Supplement: S3 Fig — Details of representative electron density and corresponding atomic models: (A) MTP in C6 reconstruction; (B) Dit, (C) Tal and (D) TMP in C3 reconstruction; (E) RBP, (F) FibL and (G) FibU in their respective focused reconstructions. (TIF) [file ppat.1008314.s003.tif]

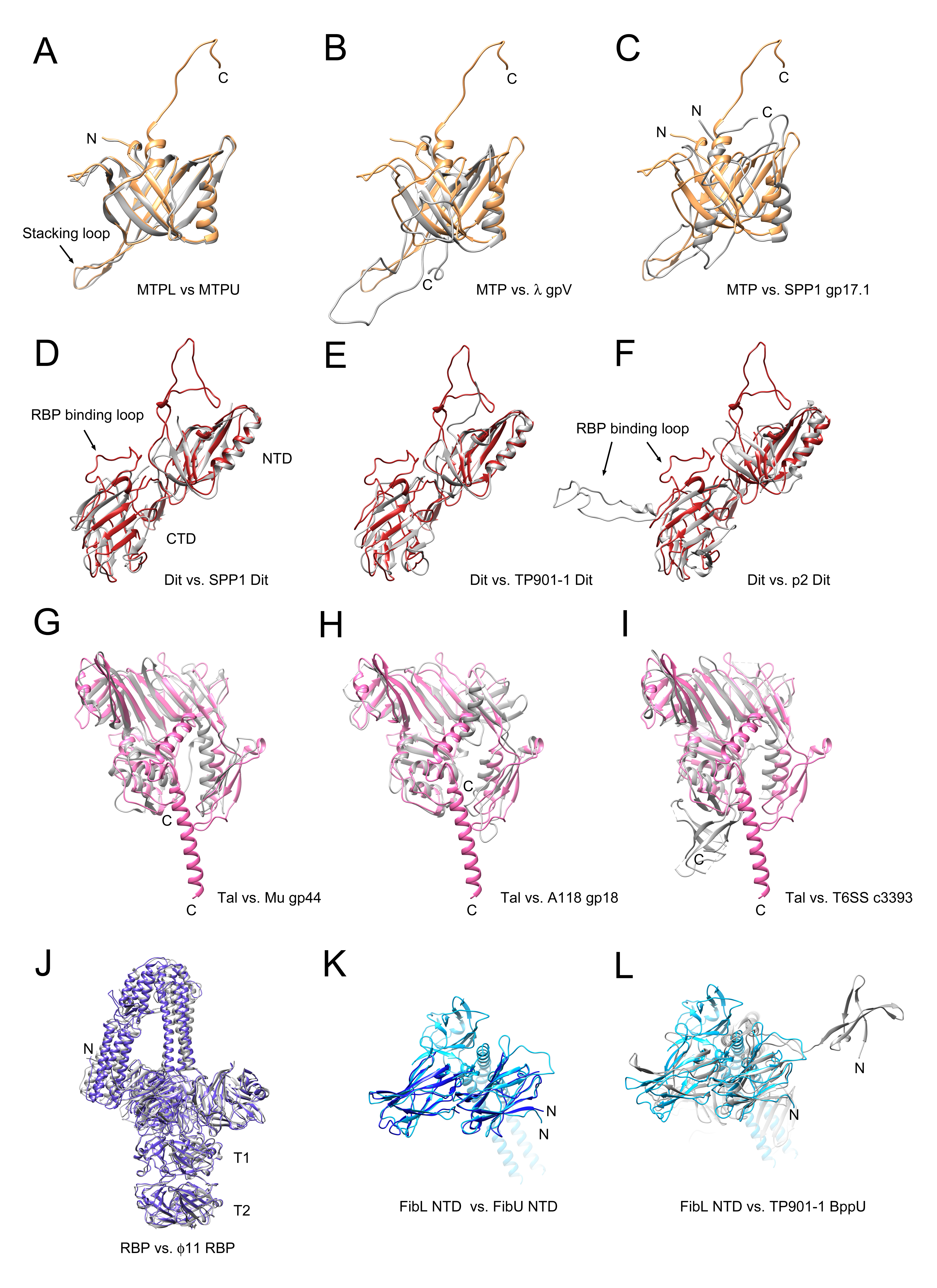

Supplement: S4 Fig — 80α proteins are colored according to the color scheme in Fig 1, while the superimposed proteins are colored gray. (A-C) MTPL (tan) superimposed on MTPU (A), phage λ gpV (B) and SPP1 gp17.1 (C). (D-F) Dit (red) superimposed on SPP1 Dit (D), TP901-1 Dit (E) and p2 Dit (F). (G-I) Tal (pink) superimposed on phage Mu gp44 (G), L. monocytogenes phage A118 gp18 (H) and E. coli T6SS protein c3393 (I). (J) RBP (purple) superimposed on ϕ11 RBP. (K-L), FibL NTD (light blue) superimposed on FibU (dark blue, K) and p2 BppU (L). Discernible N- and C- termini and relevant structure features are labeled. (TIF) [file ppat.1008314.s004.tif]

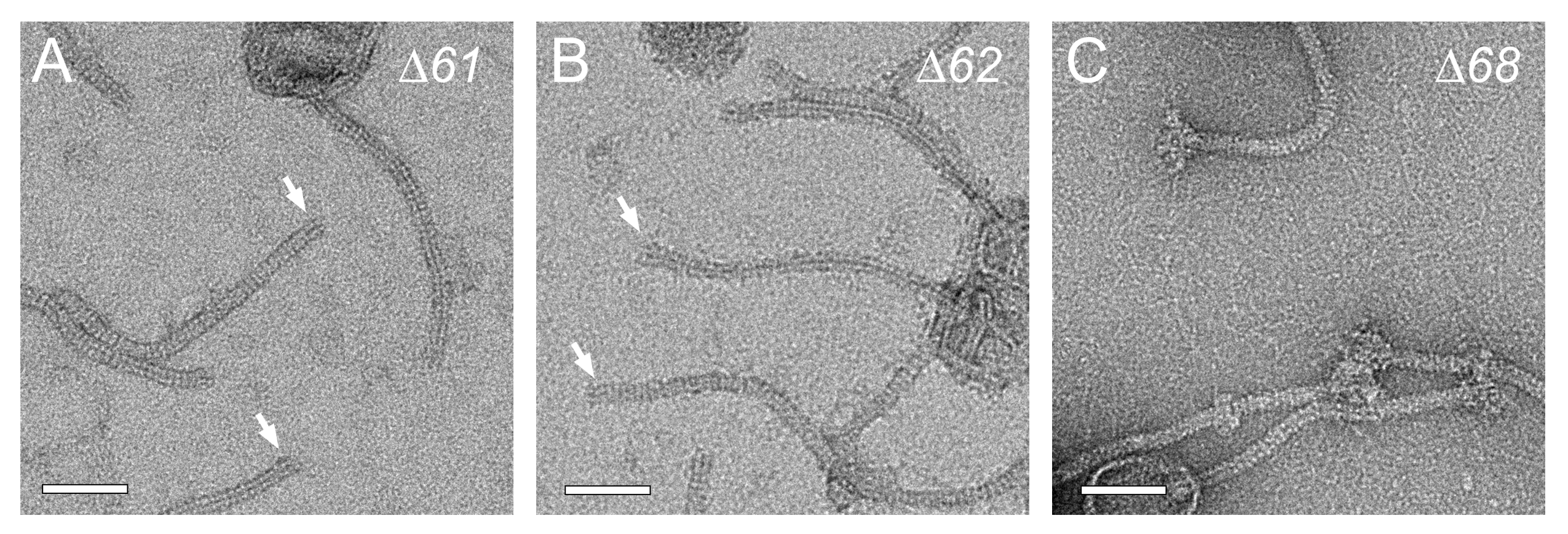

Supplement: S5 Fig — Negative stain EM of phage particles produced by 80α lysogens with deletions of ORF61 (RBP, A), ORF62 (FibL, B), and ORF68 (FibU, C). The arrowheads point to baseplates missing peripheral structures in A and B. Scale bars = 50 nm. (TIF) [file ppat.1008314.s005.tif]

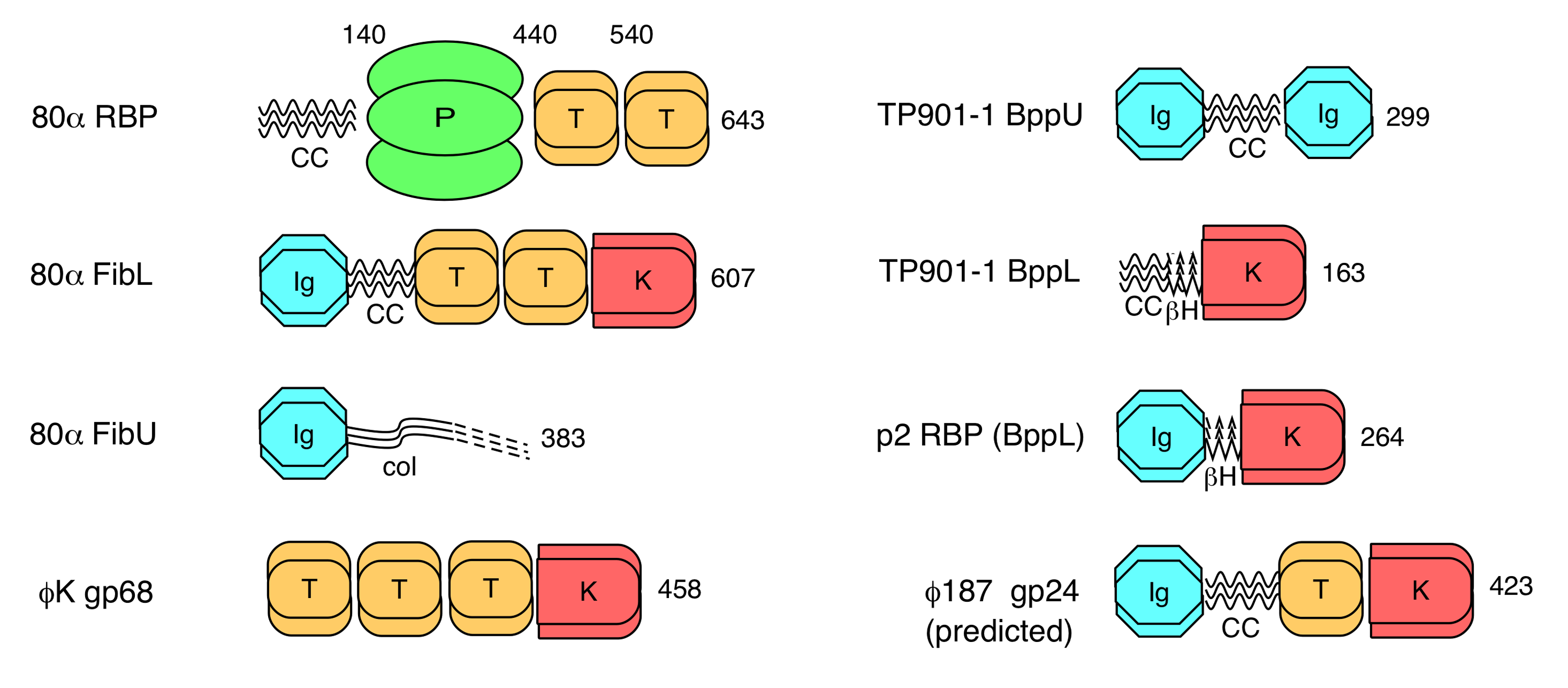

Supplement: S6 Fig — The predicted structure of the ϕ187 tail fiber is also shown. Domains are labeled: CC, coiled coil; P, β-propeller platform domain; T, tower domain; K, phage K gp68 CTD; Ig, immunoglobulin-like domain; βH, beta-helix; col, collagen-like helix. Protein lengths and relevant residue numbers are indicated. (TIF) [file ppat.1008314.s006.tif]
