# Supplementary material for: Structure of the host cell recognition and penetration machinery of a Staphylococcus aureus bacteriophage
Source: PLoS Pathog. 2020 Feb 18;16(2):e1008314. doi: 10.1371/journal.ppat.1008314 (PMC7048315; doi:10.1371/journal.ppat.1008314)
Supplement: S1 Table — The 80α reference protein and residues selected are listed in columns 1 and 2, the matched proteins and residues in columns 3 and 4. (Unless otherwise indicated, 80α proteins are implied.) The first RMSD value is between Cα atoms in all residues considered equivalent by sequence alignment. The number or residues used is listed. The second RMSD value is after pruning to remove atom pairs that are more distant than the indicated cutoff level. The number of residues compared is listed. The percentage is the fraction of equivalent residues used in the pruned set compared to the unpruned set. All data were generated using the MatchMaker function in UCSF Chimera. (PDF) [file ppat.1008314.s007.pdf]

Reference		Match		All equivalent residues		Best Matching Residues after pruning			
Protein	Res	Protein	# Res	RMSD (Å)	# Res	Cutoff (Å)	RMSD (Å)	# Res	%
MTPU	1-193	MTPL	1-193	1.50	147	2	0.71	128	87.1%
MTPL	1-193	lambda gpV	1-246	15.52	124	10	4.23	70	56.5%
MTPL	1-193	SPP1 gp17.1	1-264	13.20	148	10	5.59	91	61.5%
Dit	1-314	SPP1 Dit NTD	1-137	6.74	123	6	2.37	109	88.6%
Dit	1-314	SPP1 Dit CTD	138-253	8.53	93	6	3.45	68	73.1%
Dit	1-314	TP901-1 Dit NTD	12-147	16.33	109	6	1.90	69	63.3%
Dit	1-314	TP901-1 Dit CTD	148-253	7.04	91	6	3.99	71	78.0%
Dit	1-167	p2 Dit NTD	1-133	16.60	82	6	3.34	48	58.5%
Dit	170-314	p2 Dit CTD	134-144, 189-298	10.44	82	6	3.25	40	48.8%
Tal	2-388	*L. monocytogenes* phage A118 gp18	3-339	6.09	310	6	2.63	232	74.8%
Tal	2-388	phage Mu gp44 (1WRU)	3-346	6.55	288	6	3.24	193	67.0%
Tal	2-388	*E. coli* T6SS c3393	15-468	11.67	269	6	3.28	135	50.2%
RBP trimer	1-636	phi11 RBP trimer	1-636	5.20	1892	6	1.96	1604	84.8%
RBP trimer CTD	143-636	phi11 RBP trimer CTD	143-636	1.88	1471	6	1.75	1460	99.3%
RBP trimer CTD	143-636	P68 Tail fiber CTD trimer	151-645	18.49	1390	6	2.48	755	54.3%
RBP tower 1	445-541	RBP tower 2	542-636	2.64	270	6	2.14	257	95.2%
FibL NTD	5-169	FibU NTD	1-121	5.95	227	6	1.89	203	89.4%
FibL NTD	5-169	TP901-1 BppU NTD	1-299	12.92	328	6	2.56	204	62.2%
