# Supplementary material for: Structure of the host cell recognition and penetration machinery of a Staphylococcus aureus bacteriophage
Source: PLoS Pathog. 2020 Feb 18;16(2):e1008314. doi: 10.1371/journal.ppat.1008314 (PMC7048315; doi:10.1371/journal.ppat.1008314)
Supplement: S2 Table — (PDF) [file ppat.1008314.s008.pdf]

	Overall C6 Reconstruction	Tal-focused C3 reconstruction	RBP-focused reconstruction	FibL-focused reconstruction	FibU-focused reconstruction
**Data Collection**					
Microscope	Titan Krios 300 keV				
Detector	Direct Electron DE-20				
Pixel Size (Å)	1.21				
Defocus range (µm)	0.42-4.9				
**Reconstruction**					
Number of particles	74,294	43,601	66,861	73,384	58,716
Software	RELION-2.1.0	RELION-3.0-beta-2			
Symmetry	C6	C3	C6	C6	C6
Final resolution (FSC=0.143, Å)*	3.45	3.96	3.60	3.69	4.14
**Atomic model refinement**					
Software	PHENIX v1.15.2; *Coot* v0.8.9				
Resolution limit (Å)**	3.75	4.08	3.64	3.62	3.85
Model-map correlation (FSC=0.5, Å)	3.86	4.21	3.89	3.94	4.15
Proteins included	MTP_U and MTP_L	Dit, Tal, and TMP	RBP	FibL	FibU
Chains	12	12	24	18	12
Residues	1878	3105	11,418	3222	1464
Number of atoms					
Protein atoms	28,866	50,376	183,498	50,550	23,916
Hydrogens	13,956	25,086	89,970	25,008	12,084
Metal ions	0	0	6	0	0
Map CC_{box}	0.39	0.52	0.61	0.48	0.59
Map CC_{mask}	0.79	0.77	0.76	0.77	0.77
RMS bond length (Å)	0.008	0.007	0.006	0.002	0.002
RMS bond angles (^{o})	1.042	1.190	1.118	0.959	1.014
**Validation**					
Ramachandran plot (%)					
Favored	93.53	88.77	90.99	86.82	85.69
Allowed	6.47	11.23	8.96	13.15	14.31
Outliers	0	0	0.05	0.03	0
Rotamers outliers (%)	0	1.07	0	0.03	0
Clashscore	10.39	4.63	4.07	8.88	8.45
EMRinger score	2.68	1.39	1.49	2.19	2.57

*Between half-maps resolution reported by Phenix Comprehensive Validation after automated masking
**Between half-maps resolution reported by RELION-2/3 Postprocessing after masking and FSC correction
